# Transdermal Delivery of Chemotherapeutics: Strategies, Requirements, and Opportunities

**DOI:** 10.3390/pharmaceutics13070960

**Published:** 2021-06-26

**Authors:** Rabin Neupane, Sai H. S. Boddu, Mariam Sami Abou-Dahech, Rinda Devi Bachu, David Terrero, R. Jayachandra Babu, Amit K. Tiwari

**Affiliations:** 1 Department of Pharmacology and Experimental Therapeutics, College of Pharmacy and Pharmaceutical Sciences, The University of Toledo, Toledo, OH 43614, USA; Rabin.Neupane@rockets.utoledo.edu (R.N.); mariam.aboudahech@rockets.utoledo.edu (M.S.A.-D.); RindaDevi.Bachu@rockets.utoledo.edu (R.D.B.); davidalejandro.terrerorodriguez@rockets.utoledo.edu (D.T.); 2 College of Pharmacy and Health Sciences, Ajman University, Ajman 346, United Arab Emirates; s.boddu@ajman.ac.ae; 3 Department of Drug Discovery & Development, Harrison School of Pharmacy, Auburn University, Auburn, AL 36849, USA; ramapjb@auburn.edu; 4 Department of Cancer Biology, College of Medicine and Life Sciences, The University of Toledo, Toledo, OH 43606, USA

**Keywords:** chemotherapeutics, melanoma, breast cancer, nanoparticles, liposomes, transdermal vaccine, transdermal delivery

## Abstract

Chemotherapeutic drugs are primarily administered to cancer patients via oral or parenteral routes. The use of transdermal drug delivery could potentially be a better alternative to decrease the dose frequency and severity of adverse or toxic effects associated with oral or parenteral administration of chemotherapeutic drugs. The transdermal delivery of drugs has shown to be advantageous for the treatment of highly localized tumors in certain types of breast and skin cancers. In addition, the transdermal route can be used to deliver low-dose chemotherapeutics in a sustained manner. The transdermal route can also be utilized for vaccine design in cancer management, for example, vaccines against cervical cancer. However, the design of transdermal formulations may be challenging in terms of the conjugation chemistry of the molecules and the sustained and reproducible delivery of therapeutically efficacious doses. In this review, we discuss the nano-carrier systems, such as nanoparticles, liposomes, etc., used in recent literature to deliver chemotherapeutic agents. The advantages of transdermal route over oral and parenteral routes for popular chemotherapeutic drugs are summarized. Furthermore, we also discuss a possible in silico approach, Formulating for Efficacy™, to design transdermal formulations that would probably be economical, robust, and more efficacious.

## 1. Introduction

Presently, the global share of transdermal products in pharmaceuticals is worth billions of dollars but is limited to a few drug molecules [1]. A majority of the commercial transdermal formulations are based on small molecules (molecular weight less than 500 Da) with moderate lipophilicity (log P between 1 and 4) [2]. The advantages of transdermal delivery include the ease of drug administration and termination, avoidance of injections and hospital visits, avoidance of drug degradation by gastric pH, enzymes and hepatic first-pass metabolism, and dose-related side effects. All of these advantages make the transdermal delivery a convenient and compliant administration route for patients [3,4,5].

**Figure 1:**
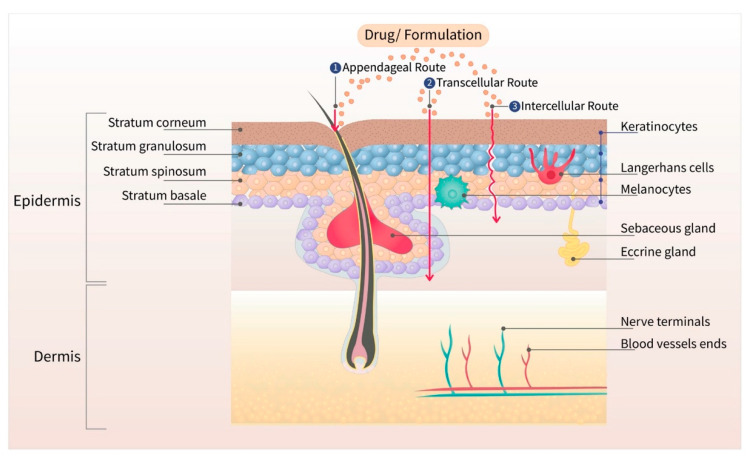
Different layers of the skin with three major routes (appendageal, transcellular, and intracellular) of drug transport. The outermost layer, stratum corneum, is the major barrier for transdermal drug delivery.

The skin is the largest organ of the body, with a barrier property that is practically impermeable to many external chemicals, microbes, and particulate matters, including colloidal components [6]. Figure 1 depicts the schematic structure of human skin and major routes of transdermal drug transport (appendageal, transcellular, and intracellular routes). Due to the lipid rich nature of the stratum corneum (SC), the outermost layer of the skin, only those drugs with moderate lipid solubility can cross the SC. Even though there are some hydrophilic channels, and pores from the sweat ducts and hair roots, only the compounds with moderate aqueous and lipid solubility can permeate across the skin for topical and transdermal delivery [7]. The barrier property of the skin widely varies depending on the body site due to the varied thickness of SC, varied distribution of pores and hair roots, and age of the patient. Due to the varied barrier property, the transdermal products are specified for application at a particular body site, such as chest, thighs, under ears, underarms, or scrotum [8,9,10,11].

The transdermal permeation of a compound is dependent on its molecular size, partition coefficient (oil–water), and surface charge [12]. Based on the porosity of the SC, only those agents with a size less than 36 nm can diffuse through lipidic or aqueous channels [13]. Based on the follicular pore size, particulates below 100 nm accumulate in the sebaceous glands and hair follicles [14,15,16]. The transdermal drug candidate should have adequate solubility in the SC lipid bilayers, which is the rate-limiting step for drug absorption. Moreover, other factors, such as the melting point, molecular weight, or molar volume, also influence the permeation of drug across the skin [17,18]. To use the transdermal route, the drug candidate should have adequate skin permeability, should be potent enough to produce therapeutic drug concentration, and cause no skin sensitization or irritation. Only, a few drug molecules are suitable for passive transdermal drug delivery. Table 1 shows the ideal properties of a drug candidate for passive transdermal delivery. All the currently approved transdermal patches, creams, and liquids are based on drugs that possess these properties. Up to the present date, there are about twenty drug molecules that have been approved for transdermal administration [19]. None of the drugs belonging to the chemotherapeutic class has been approved for transdermal administration.

Globally, cancer is the leading cause of death, and it has been estimated that 608,570 cancer deaths are expected to occur in the United States in 2021 [21]. It has been postulated that about 85–95% of cancer cases are due to exposure to carcinogenic chemicals and radiation. According to statistics published by the Skin Cancer Foundation [22], one in every three cancers diagnosed is a form of skin cancer. The main factor that predisposes people to the development of melanoma is exposure to the sun and sunburns [22]. It has been shown that the depletion of the ozone layer increases the exposure to harmful solar UV radiation, thereby increasing the incidence of skin cancer [22]. Indeed, it has been estimated that a 10% decrease in ozone levels will result in an additional 300,000 non-melanoma and 4500 melanoma skin cancer cases worldwide [22]. Melanoma is an aggressive, therapy-resistant malignancy of melanocytes that can readily metastasize into the lymphatic system, liver, and lungs [23]. It is the third most common cancer among men and women in the age range of 20–39. It has been estimated that 106,110 new cases of melanoma will be diagnosed and 7180 melanoma deaths are expected to occur in the United States in 2021 [24]. Similarly, breast cancer is the most common cancer in women in both developed and developing countries. Approximately one in four cancers diagnosed globally in women is breast cancer [25]. It was estimated that 284,200 new invasive breast cancer and 44,130 non-invasive breast cancer cases to be diagnosed in the USA in 2021 [24]. Furthermore, since 2008, the global incidence of breast cancer and mortality has increased by more than 20% and 14%, respectively [26].

**Figure 2:**
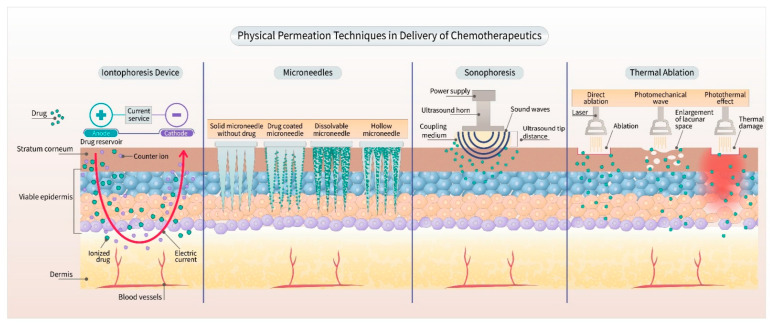
Physical permeation enhancement techniques for delivery of chemotherapeutics. Iontophoresis, microneedles, and sonophoresis are widely used approaches to enhance transdermal delivery, which relies on transient disruption of stratum corneum barrier property.

The scope of transdermal delivery of various small chemotherapeutic molecules is not fully established. The transdermal approach not only facilitates the delivery of novel therapeutics, such as small interfering (si)RNAs but also improves the efficacy in the delivery of many chemotherapeutic drugs. It is possible to use the transdermal route to treat certain types of breast and skin cancers, as the delivery of drugs can be targeted to the local tumors [27,28]. Furthermore, transdermal delivery systems can be used for the co-delivery of more than one chemotherapeutic drug for combination therapy [29]. This approach could be useful in overcoming or surmounting the drug resistance to certain anticancer drugs. In addition, the dose of drugs can be decreased in combination with synergistic effect or targeted local delivery at the tumor sites to minimize the side effects. The dose-related adverse effects can be reduced, thereby increasing the patient compliance [30,31]. Although small molecules with a log P between 2 to 4 are ideal for the transdermal formulation, hydrophilic and large molecular weight chemotherapeutic drugs can be delivered by using active enhancement methods such as sonophoresis, iontophoresis, microneedles, and laser thermal ablation (Figure 2) [32,33]. Overall, with the selection of appropriate excipients and permeation enhancement techniques, a wider range of chemotherapeutic formulations can be designed and delivered via the transdermal route.

**Figure 3:**
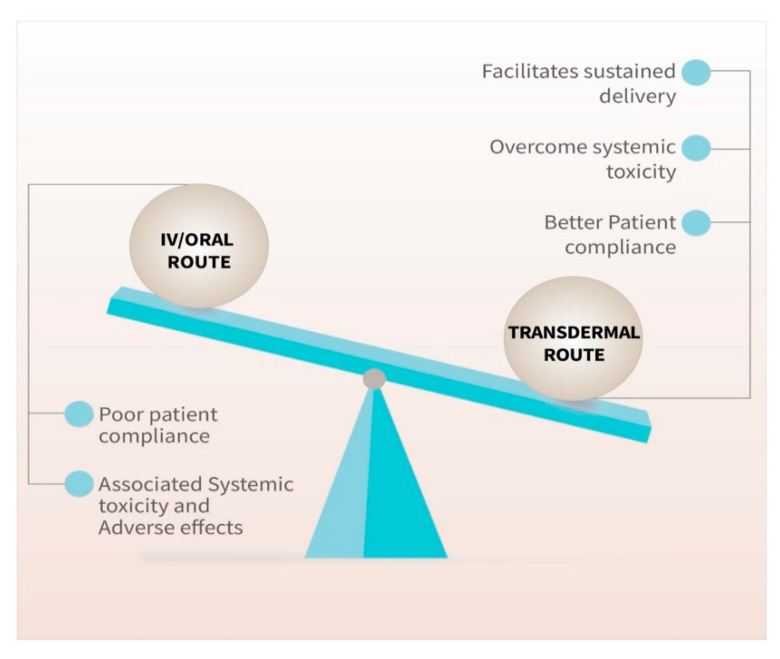
Advantages of the transdermal route over oral/IV route of delivery.

In this review, we summarize various chemotherapeutic drugs that were designed and evaluated as transdermal dosage forms for the treatment of breast and skin cancers. We also discussed the advantages of transdermal administration versus oral and parenteral delivery of various chemotherapeutic drugs (Figure 3). The design of a transdermal product by using a novel software, Formulation for Efficacy™, has been discussed. Formulation for Efficacy™ uses an in silico approach that saves time and money and provides simulations of the human skin permeation by Franz diffusion cells [34]. This approach has a higher magnitude of validity in predicting the drug permeation and diffusion of a formulation compared to the hit-and-trial approach.

## 2. Transdermal Chemotherapeutics for Breast Cancer

### 2.1. Tamoxifen Citrate

Tamoxifen citrate is available in the form of solution (2 mg/mL as a base), and tablets (10 and 20 mg as a base) for oral administration. It is a trans-isomer of a triphenylethylene derivative, with a molecular weight of 563.6 Da. It acts as a selective estrogen receptor (ER) modulator that competes with β-estradiol for the alpha-estrogen (ERα), thus inhibiting the bioactivity of estrogen in breast tissue [35,36]. It is used (1) to treat estrogen receptor-positive (ER^+ve^) metastatic breast cancer; (2) as an adjuvant to treat patients with early stage ER^+ve^ breast cancer; (3) to decrease the risk of invasive breast cancer after tumor excision and radiation in women with ductal carcinoma in situ (DCIS); and (4) to decrease the incidence of breast cancer in women at high risk [37,38]. The activity of the drug is due to the metabolites 4-hydroxytamoxifen (4-OHT) and N-desmethyl-4-hydroxytamoxifen (ENX), which are formed by the cytochrome P450 enzymes, CYP2D6 and CYP3A4/5, which have a higher affinity for ERα compared to tamoxifen [39,40].

Although the oral formulation significantly decreases the risk of recurrence of ER^+ve^ DCIS, its use can achieve the following: (1) activate ERα receptors in the endometrium (increasing the risk of cancer); (2) increase the risk of venous thromboembolic events; (3) cause vasomotor symptoms and vaginal symptoms such as dryness, discharges, and atrophy [41,42,43]. Furthermore, the efficacy of oral tamoxifen could be decreased in women with polymorphisms in CYP2D6 and CYP3A4/5 as this would decrease the levels of tamoxifen’s active metabolites [39,40]. The topical delivery of 4-OHT gel to the local tumors was shown to be a suitable alternative for oral delivery, decreasing the risk of systemic adverse effects. A randomized, pre-surgical trial in pre- and post-menopausal women was conducted by comparing transdermal 4-OHT gel (4 mg/day) to the oral tamoxifen (20 mg/day) [41]. The results indicated that equivalent concentrations of 4-OHT, i.e., 5.4 and 5.8 ng/g, were obtained in breast tissue biopsy samples from patients treated with oral and transdermal formulation, respectively. There was no significant correlation between the amount of 4-OHT in the tissue and the plasma in the transdermal formulation treated group. Thus, using the transdermal route sufficient amount of drug can be delivered to the target tissue without spiking the 4-OHT concentration in the blood. In contrast, with the oral tamoxifen group, there was a significant correlation between the concentration of 4-OHT in the plasma and the tissue leading to a spike in drug concentration, thereby causing systemic side effects. Therefore, the transdermal 4-OHT gel was found to be superior when compared to oral tamoxifen in localizing 4-OHT in patients with DCIS, thus decreasing the incidence of adverse systemic side effects, and thereby increasing the patient compliance [41]. In other study, Pathan et al. optimized a nano-emulsion of tamoxifen citrate by using arachis oil, Cremophore EL, and ethanol [44]. The solubility of tamoxifen was highest in arachis oil compared to other oils such as jojoba oil, coconut oil, castor oil and sesame oil. Among different surfactants and co-surfactants (Labrafil, Tween-80, Cremopore EL, ethanol, butanol, and propanol), Cremophore EL and ethanol demonstrated a better solubility profile for tamoxifen citrate. The optimized nano-emulsion possessed surfactant to co-surfactant ratio of 1:1, surfactant to oil ratio of 1:9, and a drug content of 5% *w/w*. This optimized formulation displayed a mean droplet size of 68 nm with a polydispersity index (PDI) of 0.125, and viscosity of 201 cP. The flux of tamoxifen evaluated by using Keshary–Chien diffusion cells across the excised rat skin was found to be 98.98 µg/cm^2^/h [44]. However, a control formulation of tamoxifen was not used to compare the efficacy of the formulation. Lin et al. [45] used bioceramic irradiation, with an emissivity of 0.98 at a wavelength of 6 to 14 μm, to enhance the transdermal delivery of tamoxifen. The permeation across cellulose acetate membrane showed that the bioceramic irradiation on water molecules weakens the hydrogen bond, which decreases the viscosity, thereby enhances the permeation. The study demonstrated higher permeation of tamoxifen and indomethacin by using bioceramic irradiation compared to the control, where bioceramics were not used [45]. Similarly, Lee et al. [46] evaluated the relative efficiency of the skin permeation of 4-OHT and ENX, using split-thickness human skin. The permeation of ENX across human skin was improved by using oleic acid as a permeation enhancer. However, the permeation of 4-OHT was significantly lower than that of ENX, even in the presence of oleic acid [46]. Dendrimer-based micelles were prepared by Yang et al. [47] to determine the feasibility of delivering ENX by the transdermal route. Generally, dendrimer-based drug-delivery systems involve the chemical conjugation of the drug with the surface groups of dendrimers to increase the drug stability during transport [48]. Due to a limited number of reactive functional groups on ENX, dendrimer conjugation was not feasible. Therefore, PEGylated dendro-based copolymers (PDCs) that can self-assemble into dendron micelles (DM) were utilized for ENX delivery [47]. The solubility and sequestration of ENX in DM was higher (3% drug loading efficiency, mean size of 48.4 nm), with smaller and uniform particle size distribution compared to the ENX cationic liposomes (0.1% drug loading efficiency, mean size of 100 nm) made with DOTAP, DMPC and cholesterol at a molar ratio of 2:2:1. Furthermore, cell titer 96 aqueous one solution (MTS) assay in ER^+ve^ MCF-7 and ER^-ve^ MDA-MB-231 breast cancer cell lines indicated that the ER-dependent, anti-proliferative efficacy of ENX was retained after encapsulation in DMs. In contrast to the liposomal formulation, the permeation of ENX from DMs across rat and human skin were sustained over 6 days. However, the intactness of the skin at the end of the study was not demonstrated. The flux of the ENX across the skin from DMs was proportional to the sequestered drug in the DMs. Despite poor drug loading, the liposomes demonstrated the highest permeability coefficient (K_p_ 0.0467 cm/h). Moreover, the DMs (size >24 nm) cannot permeate through the aqueous pores of the skin (size <4 nm), as they have a significantly larger size compared to the aqueous pores of the skin. However, DMs facilitate the permeation of drugs through the skin by translocating the drug molecules [47].

**Figure 4:**
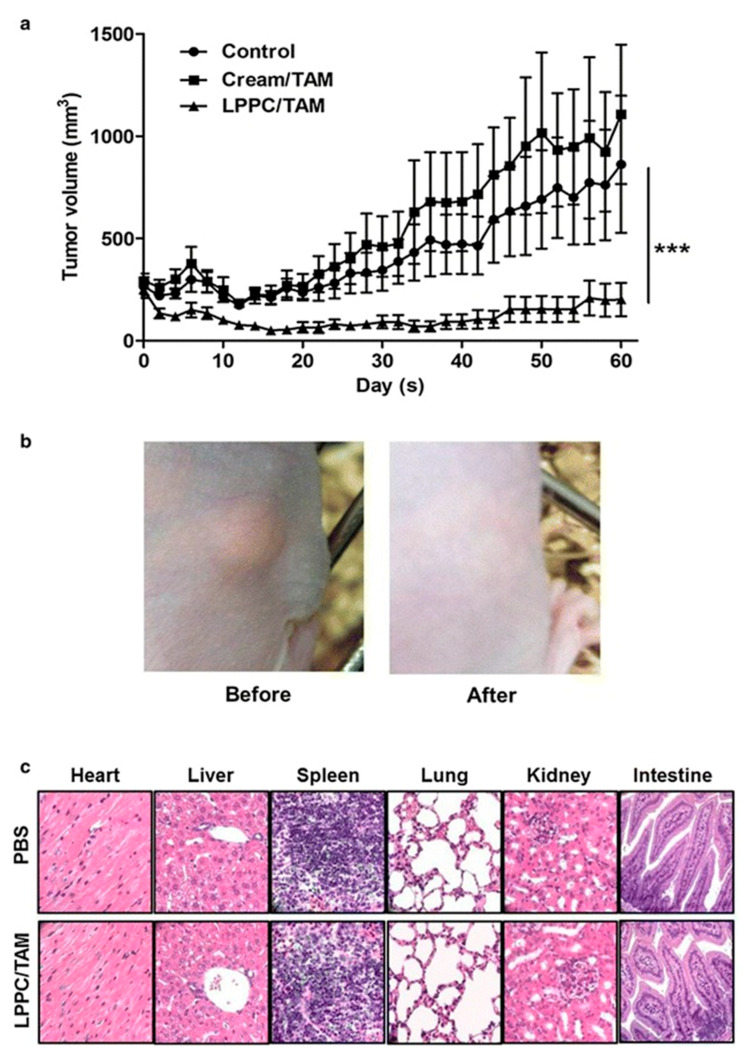
Antitumor efficacy of liposome–PEG–PEI complex/Tamoxifen (LPPC/TAM) in BT474-tumor-bearing mice via transdermal treatment. (**a**) Following transdermal application of the cream/TAM or LPPC/TAM to the tumor area every day, the tumor volume was measured with a caliper, and was calculated as L × H × W × 0.5236. The animals were sacrificed after 60 days of implantation of the 60-day release 17β-estradiol pellet (*n* = 5). (**b**) Observations of the skin in the tumor-bearing mouse before and after treatment with LPPC/TAM. (**c**) Histopathological evaluations of the heart, liver, spleen, lungs, kidneys, and intestines in the LPPC/TAM treatment group. Sections of the tissue were fixed in 10% formaldehyde overnight, embedded in paraffin and were cut into slices. Later, tissue sections were stained by using H&E. *** *p* < 0.001. Reproduced with permission from Lin et al. [49].

Lin et al. [49] used the liposome–PEG–PEI complex (LPPC) for the transdermal delivery of tamoxifen in vivo in mice xenograft with BT474 breast cancer cells. The in vitro efficacy of the LPPC-tamoxifen was determined in the breast cancer cell lines, ER^+ve^ MCF-7, DT474 and ER^-ve^ MDA-MB-231, using the 3-(4,5-dimethylthiazol-2-yl)-2, 5-diphenyl tetrazolium bromide (MTT assay). Although the IC_50_ values were not determined, the liposomal formulation significantly reduced the viability of the ER^+ve^ and ER^-ve^ cell lines. Tamoxifen arrested the proliferation of ER^-ve^ breast cancer cell lines in the S phase of the cell cycle. Furthermore, the study showed that the inhibition of the CIP2A/PP2A/p-Akt (Protein phosphatase 2A (PP2A), a cellular inhibitor of PP2A/CIPP2A, protein kinase B-Akt) signaling pathway was responsible for the inhibition of ER^-ve^ cell proliferation. The LPPC-tamoxifen (LPPC/TAM) decreased tumor growth by 82% compared to the control. LPPC/TAM was also found to effectively inhibit BT474 tumor growth than cream-tamoxifen. In addition, the tissue damage and pathology of organs induced by LPPC/TAM treatment were assessed. The results in the treated mice showed that LPPC/TAM did not cause any skin irritation or injury to organs (Figure 4). Overall, the results indicated the feasibility of using LPPC formulations for the local delivery of tamoxifen in breast cancer cells in an in vivo mouse model [49]. Several research studies demonstrated that local delivery of tamoxifen and its derivatives via transdermal route is possible. Moreover, the compounds disperse in a gel matrix or in the form of nanomedicines (ethosomes, liposomes, dendrimers, etc.) and can provide effective drug levels at the tumor sites in animal models.

### 2.2. Letrozole

Letrozole is available as 2.5 mg tablets for oral administration. It is a dibenzo nitrile derivative, which has a molecular weight of 285.3 Da and a log P of 1.27. It is used in the treatment of estrogen-dependent breast cancer. It is a reversible inhibitor of the enzyme aromatase, which catalyzes the last step in the synthesis of estrogen, thereby blocking the production of estrogen [50,51]. The oral formulation of letrozole significantly decreases the plasma levels of estrogen [50]. However, estrogen levels in local tissue, such as the breast, can be significantly greater than in the plasma [50,52,53]. Furthermore, the depletion of circulating estrogens by letrozole can produce vasomotor symptoms and adverse effects on the bone tissue [52,53].

The physicochemical properties and the low dose of letrozole are very favorable for designing a transdermal formulation. Li et al. [54] determined the concentration of letrozole in plasma, skin, and breast tissue of mice that received oral suspension (50 mg/kg) or transdermal patch containing 3 mg/5cm^2^ of letrozole. Following the oral administration, letrozole level in breast tissue and plasma were 0.15–2.38 μg/g and 0.20–4.80 μg/mL, respectively, whereas, after transdermal administration, the letrozole level was 10.4–49.3 μg/g, 1.6–6.8 μg/g, and 0.35–1.64 μg/mL in the skin, breast tissue, and plasma, respectively. The oral delivery showed elevated plasma concentration of letrozole compared to the skin. Overall, the transdermal delivery of letrozole was more efficient, which showed localized delivery to the breast tissue instead of elevating the plasma concentration of the drug. Moreover, the low systemic levels of letrozole from the patch could decrease the incidence of the aforementioned adverse effects [54]. Li et al. [55] optimized the delivery from the transdermal patch of letrozole by using various adhesives, permeation enhancers, and different letrozole concentrations. The permeation of letrozole through excised rat skin was significantly higher through the patch with adhesive DURO-TAK^®^ 87-4098, which lacks a carboxyl group, compared to DURO-TAK^®^ 87-2677 and 87-2852, the adhesives with a carboxyl group. It was speculated that the triazole group of letrozole could interact with the carboxyl group to form hydrogen bonds [55], which could impede the release of the drug from the adhesive with carboxylic groups. Conventional chemical enhancers were used to optimize the permeation, and the results indicated that a combination of azone (10%) with propylene glycol (5%), containing 1.5% of letrozole, was optimal for the adhesive patch [55]. Maniyar et al. [56] formulated spray-dried letrozole (SPD-LET) as a liposomal dispersion in cream for topical delivery to breast cancer tumors. The liposomal cream, which contained peppermint oil, produced a greater permeation than olive oil. In vitro experiments using MTT assay indicated that the SPD-LET formulation had superior anti-proliferative activity with lower viability in MDA-MB-231 (breast cancer) cell line compared to the group treated with plain letrozole cream. Moreover, the in vivo pharmacokinetic profile in Wistar rats was compared between the plain letrozole cream and SPD-LET cream. The C_max_, T_max_, and AUC were 11.3 μg/mL, 3 h, and 101.7 μgh/mL for SPD-LET cream, whereas 4.2 μg/mL, 5 h and 37.8 μgh/mL for plain letrozole cream. Overall, the pharmacokinetic profile of the letrozole liposomal cream was superior to that of plain letrozole cream. It was assumed that the SPD liposomes contain lipid components that get adsorbed on the SC and progressively merge into the polar lipids which enhances the drug delivery across the skin [56]. The existing literature suggest that letrozole formulated in the form of a transdermal patch or a liposomal cream are effective to deliver the drug across the skin into the local tumor tissue, and into the systemic circulation. Since letrozole tablets induce nausea, vomiting, and hot flashes, transdermal administration could potentially be an alternative to overcome the side effects.

### 2.3. Anastrozole

Anastrozole is available as 1 mg tablets for oral administration. It is a benzenediacetonitrile derivative and has a molecular weight of 293.4 Da. It binds to the cytochrome P-450 component of aromatase and reversibly inhibits its activity [57]. Although anastrozole and letrozole belong to the same class of aromatase inhibitors (triazoles that are reversible aromatase inhibitors), they differ in their pharmacological profile (letrozole seems to be more potent compared to anastrozole) [57]. Similar to letrozole, the physicochemical and pharmacological properties of anastrozole are very favorable for designing a transdermal formulation. Xi et al. [58] designed an adhesive matrix transdermal patch of anastrozole, using DURO-TAK^®^ 87-4098, adhesive without the carboxylic group, and 8% isopropyl myristate (IPM). Anastrozole also contains a triazole moiety as letrozole, which is a hydrogen bond donor and acceptor. It was observed that DURO-TAK^®^ without carboxylic groups showed higher drug delivery (3.84 times higher) compared to the DURO-TAK^®^ adhesive patch with a carboxylic group. Among the different penetration enhancers evaluated (i.e., Transcutol^®^, IPM, oleic acid, and l-menthol), IPM at a concentration of 8% produced the highest flux (26.13 ± 6.75 μg/cm^2^/h) of anastrozole across the excised rat abdominal skin. An in vivo study was conducted in mice comparing the oral suspension (15 mg/kg) and transdermal patch (2 mg/cm^2^) of anastrozole, where the concentration of drug in skin, muscles, and plasma were quantified at different time points. Anastrozole delivered by transdermal route localized in the skin and muscles as a local depot providing sustained plasma level for 12 h, whereas the oral anastrozole was absorbed systemically and reached a peak plasma concentration within 1 h. The muscle–plasma concentration ratios were 49.06, 43.02, 26.91, 41.48, and 51.29 at a time point of 0.17, 1, 4, 8, and 12 h via transdermal route while the ratios were 0.79, 077, and 1.09 in 0.17, 1, and 4 h via oral route. The results indicated that a sustained delivery of anastrozole from the patch to the skin and surrounding tumors could be achieved [58]. Regenthal et al. [59] formulated a transdermal patch of anastrozole and compared its pharmacokinetic profile in dogs with the PK results obtained by Mende et al. [60], using the oral anastrozole formulation (tablets) in human subjects. Silicon matrix BIO-PSA^®^ type 7-4302, ethyl acetate as a solvent, glycerol as crystallization inhibitor, and CoTran™ 9720 backing film were used in the transdermal patch. The transdermal patch produced a rapid and linear delivery of anastrozole in beagle dogs within the first 24 h (C_max_ = 5.8 ng/mL), and after reaching a plateau, there was a slow decrease in the next two days [59]. The oral tablets of anastrozole produced a rapid, maximal concentration, whereas the transdermal patch produced a steady release of the anastrozole. The area under the curve (AUC) for the transdermal anastrozole patch was comparable to the oral formulation, but the half-life of anastrozole increased 2-fold following the application of the transdermal patch [59]. The state of the drug in the patch (i.e., homogeneity and crystallinity) significantly affects the drug-release pattern from the patch. It was also shown that ethyl acetate was superior over other solvents such as THF, DMSO, xylene, ethanol, dioxane, chloroform, and dichloromethane, owing to its higher drug solubility and compatibility with the adhesives [59]. Unlike oral formulations of anastrozole the transdermal patch does not cause a spike in the plasma drug levels, thereby avoiding the adverse effects associated with it.

## 3. Transdermal Chemotherapeutics for Melanoma

### 3.1. Imatinib Mesylate (IM)

Imatinib mesylate (IM) is a benzamide methanesulfonate derivative, which has a molecular weight of 589.7 Da. It is available as tablets (100 mg and 400 mg) for oral administration. IM is used to treat patients with certain types of chronic myelogenous leukemia (CML). It binds in an area in the BCR-ABL protein that binds its substrate, ATP, and inhibits the catalytic transfer of a phosphate group to a specific tyrosine, thus inhibiting the phosphorylation of certain proteins that mediate the pathophysiology of CML. In addition, imatinib inhibits the protein, c-kit, a Type III receptor tyrosine kinase (also known as CD117) that is activated by the endogenous proteins, stem cell factors (SCFs), present in certain types of tumors [61]. The overexpression of c-kit receptors in melanoma increases the expression of the microphthalmia-associated transcription factor and downregulates the anti-apoptotic protein, Bcl2, thereby promoting cancer progression [62].

Although IM is highly lipophilic (log P 4.38), and has a high dose, researchers have attempted transdermal formulations for this drug by using carrier systems such as nanoparticles and active permeation techniques. Gold nanoparticles (AuNP) have been utilized for the delivery of different anticancer molecules, such as cetuximab, gemcitabine, cisplatin, etc., primarily by conjugating the drug to the surface of the particles [63]. Filon et al. have used both intact and damaged human skin to demonstrate the route of delivery of nanoparticles following transdermal application is typically paracellular or appendageal [64,65]. For the delivery of IM, Labala et al. [66] used positively charged polymers, polyethylene imine (PEI), and polystyrene sulfonate (PSS), to coat the gold nanoparticles by using a layer-by-layer strategy that produced stable gold nanoparticles without a significant increase in the particle size. IM was loaded on gold nanoparticles for active transdermal delivery, i.e., iontophoretic transport system. Moreover, the typical features of layer-by-layer polymer-coated AuNPs, such as small particle size (98.5 nm), high surface-charge density, and positive charge (32.3 mV), in combination with iontophoresis (0.47 mA/cm^2^ for 4 h), make them a suitable candidate for permeation across the skin [66]. The loading efficiency of IM was 28%, which was most likely released via a diffusion-controlled mechanism from the NPs. The permeation of IM-loaded AuNPs in conjunction with iontophoresis was 6.2-fold higher compared to the passive application. Moreover, in the cytotoxicity assay, the IM containing nanoparticles produced 80% inhibition of B16F10 melanoma cells at a concentration of 77.5 μM [66]. However, the formulations containing PEI should take cytotoxicity of PEI into account which could be associated with its high density of cationic charge and can also vary depending on the cell line [67]. In another similar study, Labala et al. [68] used a combination anticancer-drug approach, consisting of signal transduction and activator of transcription factor 3 (STAT3) siRNA with IM in AuNPs coated with multiple layers of chitosan. The chitosan provides a layer of positive charge, where the negatively charged siRNA could be sandwiched, and IM encapsulation solely depended on electrostatic interactions and hydrogen bonding, which enhanced the efficiency of drug loading. The dual drug-loaded nanoparticles, at 130 µM, significantly inhibited the growth of B16F10 melanoma cells in vitro. The in vivo efficacy of the nanoparticles was determined by using C57BL/6 mice with B16F10 melanoma cells via transdermal iontophoresis and intra-tumoral routes. The reduction of the tumor weight and volume following treatment via intra-tumoral route and transdermal route (iontophoresis: 0.5 mA/cm^2^ for 2 h) were not statistically significant. This result demonstrated comparable efficacy between the iontophoretic treatment and the intra tumoral delivery. To validate the molecular basis of successful delivery of the STAT3 siRNA, the levels of STAT3 expression were determined by using Western blots. The expression levels of STAT3 were significantly decreased with the transdermal (iontophoretic) administration of siRNA-IM AuNps compared to control. However, the drug loading in NPs must be further improved to ensure maximal delivery by using minimal NPs. The use of other human skin cancer cell lines, along with molecular markers indicative of cytotoxicity, would help to further validate the development of such novel delivery systems [68]. In summary, IM showed promising results in a nanoparticle based transdermal formulation. These studies indicate the possibility to deliver molecules via transdermal route even if the molecule needs to be delivered at a higher dose or has high lipophilicity.

### 3.2. Vemurafenib

Vemurafenib is a propane sulfonic acid derivative and has a molecular weight of 489.9 Da. It is available as tablets (240 mg) for oral administration. It is an inhibitor of the kinase activity of the BRAF kinase that has the valine to aspartate mutation at position 600 (i.e., the BRAF(V600E) kinase) [69], which is present in at least 60% of all melanomas [70]. Clinical studies indicate that the oral formulation of vemurafenib can produce hepatic and renal toxicity [71,72,73,74]. Vemurafenib is specifically approved for the treatment of metastatic melanoma. Zou et al. [74] evaluated the efficacy of the transdermal vemurafenib delivered via peptide-modified liposomes, using an in vivo mouse xenograft model. The peptide TD (ACSSSPSKHCG) was used as a permeation enhancer that transiently opens the paracellular pathway of the skin, thereby facilitating drug permeation [75]. To assess the toxicity profile of the liposomal content, the viability of A375, B16F10, and HUVEC cells were determined after treating those cell lines with blank liposomes for 72 h followed by MTT assay. Viability greater than 95% was reported which indicated the non-toxic nature of liposomal content and permeation enhancer. The in vitro permeation study carried out by using rat abdominal skin showed significantly higher permeation of vemurafenib from peptide-modified liposomes compared to liposomes without TD. For safety study BALB/c mice were given liposomal formulation every two days, for 7 days, at a dose of 0.25 mg of vemurafenib via three different routes, i.e., tail vein, oral gavage, and transdermal (abdominal region). Histopathological evaluation of kidney, heart, lungs, and liver showed significant damage to liver, lungs, and kidney in animals treated via oral and intravenous route, but such toxicity was not observed in the group treated via transdermal route. The in vivo study was carried out using xenograft model (BABL/c nude male mouse injected subcutaneously with BRAF mutant A375 cells) in which the liposomes containing 0.25 mg of vemurafenib was administered every 2 days, for 18 days, via three different routes oral, tail vein, and transdermal (daubed at the site of tumor). The tumor weight and tumor volume suppression in the group treated via transdermal route were much smaller compared to the other routes (oral and intravenous routes) [74]. This study exemplifies the possibility to overcome the oral toxicity of vemurafenib by using transdermal administration as an alternative route. Even though the oral dose is high, vemurafenib can still be delivered topically to the tumor regions to avoid undue systemic exposures.

### 3.3. Five-Aminolevulinic Acid (5-ALA) Hydrochloride

Chemically, 5-Aminolevulinic acid (5-ALA) hydrochloride is 5-amino-4-oxo-pentanoic acid hydrochloride with a molecular weight of 167.59 Da. It is available in the form of a lyophilized powder for oral solution (30 mg/mL), topical gel (10%), and topical solution (20%). Moreover, 5-ALA is an FDA-approved photodynamic therapy (PDT)—based drug used for the targeted therapy of cutaneous T-cell lymphoma, basal cell carcinoma, and squamous cell carcinoma [76,77]. PDT is based on the biotransformation of a prodrug to its active form, using light irradiation [78]. Moreover, 5-ALA is bio-transformed to protoporphyrin IX (PpIX) following irradiation with light, at a wavelength of 635 nm, which induces the formation of reactive oxygen species, resulting in tumor cell death [79]. Although 5-ALA is a small molecule, its permeation across the skin is limited by its hydrophilic nature. In order to overcome this limitation, Pierre et al. [80] prepared liposomes of size 400 nm, consisting of ceramides (50%), cholesterol (28%), palmitic acid (17%), and cholesteryl sulfate (5%), that had a similar composition to the mammalian SC to increase the delivery of 5-ALA across the skin. As expected, only 5.7% of the drug was encapsulated in the liposomes as 5-ALA is very hydrophilic in nature. The in vitro permeation study was carried out by using freshly excised rat dorsal skin over 36 h. The total amount of drug permeated and flux across the skin was higher for aqueous solution (3681 ± 104.65 μg and 38.3 ± 2.4 μg/cm^2^h) compared to the liposomal formulation (500.9 ± 32.5 μg and 4.2 ± 0.2 μg/cm^2^h). However, the amount of 5-ALA retained in the dermis and the epidermal layer was significantly higher for the liposomal formulation of 5-ALA compared to the control (5-ALA solution). Thus the liposomal formulation helps in localizing the drug around the application site [80]. Lin et al. [81] used 1,2 dipalmitoyl-sn-glycero-3-phosphocholine (DPPC) to produce a liposomal formulation of 5-ALA to further increase the entrapment efficacy of the drug in carrier systems to 15–16%, where the liposome size was 100 nm. In vitro, cytotoxicity assay was carried out in B16F10 melanoma cells, where a light dose of 50 J/cm^2^ was used over 20 min after liposomal treatment. The viability of the cells was 52% after treatment with 5-ALA liposomes without DPPC whereas the viability was 33% with 5-ALA-DPPC liposomes. Further, the mitochondrial membrane potential was significantly reduced, and intracellular ROS levels significantly increased in 5-ALA-DPPC-treated cell lines compared to the 5-ALA liposomes without DPPC. In a mouse xenograft tumor model (B16F10 cell implanted subcutaneously), the tumor volume was significantly smaller in those treated with liposomes of 5-ALA compared to control. However, there was no significant difference in tumor volume in 5-ALA liposome treated, and 5-ALA/DPPC-treated group. Nevertheless, the level of Protoporphyrin IX (PpIX) in the tumor was significantly higher in 5-ALA/DPPC liposome treated group compared to the 5-ALA liposome group indicating the DPPC enables better permeation of drug across the skin to reach the tumor site [81]. In summary, 5-ALA is a hydrophilic drug that has been used for PDT and researchers made attempts to deliver this drug to the tumor sites as a liposomal formulation. Due to poor entrapment efficiency of the liposomes, and the larger dose requirements, currently, there are no effective formulations for topical delivery of 5-ALA. Ethosomes and niosomes are promising delivery systems for hydrophilic compounds [82] which could serve as potential carriers to deliver 5-ALA across the skin to the tumor sites.

## 4. Plant Product–Based Transdermal Chemotherapeutics

### 4.1. Curcumin

Curcumin is a polyphenolic constituent of turmeric powder that has various health benefits and antitumor efficacy [83]. It has been postulated that curcumin’s anticancer efficacy could be due to the induction of apoptosis, inhibition of of certain intracellular transcription factors and the downregulation of various secondary messengers, COX2, c-Jun, nitric oxide synthase, and matrix metalloproteinase-9 [84]. Curcumin is poorly soluble in water leading to poor absorption, which is one of the reasons for its low oral bioavailability. The other reasons include high instability, rapid metabolism, and rapid systemic elimination; all of these contribute to low oral bioavailability [85]. Lee et al. [86] summarized the nano-formulation strategy for curcumin and the limitations related to its use as an anticancer therapy. Here, we primarily focus on discussing recent publications that specifically report using transdermal formulations for the delivery of curcumin. Sun et al. [87] used hydroxypropyl-β-cyclodextrin (HP-β-CD)-curcumin complexation approach to improve the solubility and stability of curcumin. The grinding method was used for complexation where the molar ratio of curcumin to HP-β-CD was 1:2. An inclusion efficacy of 97.4% was achieved. The complex was further formulated as a hydrogel with poloxamers 407 and 188. This study demonstrated a 20-fold increase in the water solubility of curcumin along with higher photostability. The inclusion complex preserves the labile phenol hydroxyl group from degradation. The viability of B16F10 cells after the treatment with curcumin hydrogel (300 μg/mL) and curcumin-inclusion-complex hydrogel (300 μg/mL) was 83.1% and 15.12% respectively. The higher efficacy of the curcumin inclusion hydrogel was attributed to the enhanced solubility of the inclusion complex in the hydrogel [87]. Jose et al. [88] used deformable cationic liposomes in combination with iontophoresis to co-deliver curcumin and STAT3 siRNA. STAT3 is an oncogenic transcription factor that is overexpressed in various types of cancer, including melanoma [89]. Its transcriptional activity can be inhibited by small interference RNA (siRNA) [90]. However, there are significant challenges associated with the delivery of siRNA due to its poor in vivo stability and penetration across the cell membrane barrier [91]. The uptake of liposomes was studied in A431 cells with or without the endocytosis inhibitors (Chlorpromazine hydrochloride and methyl-β-cyclodextrin). The uptake was reduced when the inhibitor was used compared to the cells without the endocytosis inhibitor indicating the uptake occurred via Clathrin and caveolae pathway. MTT assay was carried in A431 cells where the combination of STAT siRNA and curcumin liposomes (with 250 µM curcumin and 0.5 nM STAT siRNA) showed highest growth inhibition of 72.9% compared to curcumin liposomes (350 µM) and SiRNA liposomes (1 nM), which showed inhibition of 32.2% and 56.9% respectively. Further iontophoresis, at a current density of 0.47 mA/cm^2^ for 4 h, was used to enhance the permeation of liposomes across the porcine ear skin. The group demonstrated 5-fold higher deposition of the curcumin in the skin by using iontophoresis compared to passive permeation of the liposomes suggesting transdermal delivery of the complex is feasible [88].

Several studies suggest that curcumin could target different pathways associated with breast cancer, which can be useful in the treatment of certain types of breast cancer [92]. Recently, studies have been conducted to develop transdermal delivery of curcumin to the breast tissue. Atlan et al. [93] proposed the fabrication of disposal bra inserts for the transdermal delivery of curcumin. Since curcumin is clinically safe up to doses of 8 g/day [93], they proposed the use of transferosomes to deliver curcumin in bra inserts as a preventive measure against breast cancer. The authors postulated that a preventive regimen of curcumin would modulate inflammatory biomarkers, limit ROS-induced damage to existing breast cells and eliminate incipient abnormal cells before they proliferate, thereby reducing the incidence of breast cancer [93]. Abdel-Hafez et al. [94] evaluated the effect of the penetration enhancers (Labrasol^®^, Transcutol^®^, limonene, and oleic acid) on the permeation of curcumin transferosomes by using phosphatidylcholine across the dorsal and abdominal excised skin of mice. The flux of curcumin from the oleic acid (15.058 μg/cm^2^h) and Transcutol^®^ (15.678 μg/cm^2^h) formulations was higher compared to the flux of curcumin from Labrasol^®^ (10.266 μg/cm^2^h) and limonene (10.189 μg/cm^2^h) [94]. Pushpalatha et al. [95] formulated nano-sponges of cyclodextrin, using pyromellitic dianhydride as a crosslinker. The nano-sponges were loaded with curcumin and resveratrol and were dispersed in a carbopol gel for transdermal delivery. The permeability study across porcine ear skin indicated that the hydrogel with nano-sponges produced a 10-fold and 2-fold higher permeation of curcumin and resveratrol, respectively, compared to hydrogel formulation without nano-sponges. The nano-sponge formulation also produced a 7-fold increase in the photostability of curcumin and resveratrol compared to hydrogel without nano-sponges. MTT assay in MCF-7 cells, treated with a combination of curcumin and resveratrol nano-sponges in the ratio of 1:1 and 1:3 resulted in IC_50_ of 15 µg/mL and 10 µg/mL, respectively [95]. All of these studies indicate that a suitable formulation design not only enhances the transdermal permeation but also promotes drug stability especially of those drugs, which are photosensitive such as curcumin.

### 4.2. Resveratrol

Resveratrol is a phenolic antioxidant present in natural foods, such as grapes, wine, berries, nuts, etc. [96]. Numerous studies suggest that resveratrol has anticancer activity in skin, breast, lung, liver, prostate, colon, and ovarian cancers [97]. However, the pharmacokinetic profile of resveratrol is not ideal for therapeutic use. Resveratrol is well absorbed orally by passive transport (~75%), but it undergoes extensive hepatic metabolism by glucuronidation and sulfate conjugation. In addition, resveratrol forms complexes with the low-density lipoproteins, plasma proteins such as albumin, leaving less than 1% resveratrol in the systemic circulation [98]. Therefore, there is a need for the development of bioavailable and efficacious formulations of resveratrol.

Resveratrol, by virtue of its antioxidant properties prevents UV-induced skin damage. In the skin, it inhibits lipid peroxidase and activation of NFkB [99]. Resveratrol skin treatment pre- and post-exposure to UVB light dramatically reduced the skin damage and skin cancer occurrence [100]. In addition to chemo-preventive effects, resveratrol also inhibits tumor progression by suppressing the growth of skin cancer by inhibiting DNA polymerase and deoxy-ribonucleotide synthesis and inducing cell-cycle arrest [101]. Tsai et al. [102] optimized and evaluated the potential of nanostructured emulsion carriers composed of isopropyl myristate or caproyl 90 with different surfactants (Brij 35, Tween 80, and L44) for transdermal delivery of resveratrol. IPM was used as an oily phase. The optimized nanostructured emulsion (size 277 nm and viscosity 5.82 cps) contained IPM (HLB 11.1) with Tween80/Span20 (with an HLB value of 11.16). The skin permeation and deposition of resveratrol across excised rat skin at the end of 24 h from saturated aqueous resveratrol solution (control) were 0.79 ± 0.78 and 4.26 ± 0.58 μg/cm^2^ whereas it was 276 ± 42.3 and 29.4 ± 7.7 μg/cm^2^ when optimized nano-emulsion consisting IPM with Tween80/Span20 was used. There was an 896.2-fold increase in drug permeation and a 10.2-fold increase in skin deposition with the use of nano-emulsion. Bioavailability experiments indicated that orally administered resveratrol suspension (dose 30 mg/kg) was rapidly metabolized and eliminated from the blood within 10 h of administration. In contrast, the transdermal administration of resveratrol (dose: 67 mg/kg applied on the shaved abdomen) produced a steady and prolonged level of resveratrol in the blood with C_max_ around 25 h [102]. Similarly, Hu et al. [103] used a non-aqueous, self-double-emulsifying drug delivery system (SDEDDS) for transdermal delivery of resveratrol. SDEDDS is based on the principle that the drug is highly soluble in the innermost oily layer but less soluble in the outer oily layer, followed by the layer of surfactants [104]. Solubility of resveratrol in different organic phases (PEG400 < Transcutol^®^ CG < propylene glycol < ethanol) and natural oils were reported (olive oil < evening primrose oil < aloe oil < avocado oil < grape seed oil < soybean oil < corn oil < coconut oil.) Evening primrose oil, Polyglycerol polyricinoleate (PGPR) as a hydrophobic surfactant (8%), Tween 60 (4%) as a hydrophilic surfactant and organic phase constituting PEG400, propylene glycol, and Transcutol^®^ were used in the optimized formulation. The optimized formulation produced a biphasic release of the resveratrol, i.e., a burst release followed by a controlled release. The biphasic release was due to distribution of the drug between the o/o emulsion and the hydrophilic surfactant during homogenization. Although resveratrol was initially solubilized in the innermost oily layer, the drug is distributed in the outer most layer of the hydrophilic surfactant during homogenization. The burst release of resveratrol is due to the release of the drug from the outer layer. In contrast, resveratrol in the inner oily layer was released in a sustained manner. In vitro permeation across porcine ear skin showed 8.3 fold higher flux and 10 folds higher skin deposition compared to the aqueous solution of resveratrol [103]. Park et al. [105] used chitosan-coated liposomes to enhance the skin permeation of resveratrol. An in vitro permeation across the full thickness of dorsal mouse skin with chitosan-coated liposome (containing 0.1% resveratrol) showed a 126.93 μg/cm^2^ (40.42%) permeation of resveratrol over 24h. The uncoated liposomes showed 96.85 μg/cm^2^ (30.85%) permeation. Chitosan imparts a positive charge to the liposomes, which interact with the negative charge of the SC, thereby facilitating the permeation of the drug carrier system [105]. Pentek et al. [106] developed a dendrimer–resveratrol complex by using fourth generation polyamidoamine (PAMAM) dendrimers. This formulation increased the solubility and stability of resveratrol in aqueous solution and semisolid dosage forms (cream). The in vitro permeation study carried out by using rat skin showed higher permeation, using dendrimers, as compared to aqueous solution of the resveratrol. The advantage of using a PAMAM dendrimer compared to the liposomal preparation is that they are devoid of organic solvents and oils that can be irritating or toxic to the skin [106]. Carletto et al. [107] formulated polycaprolactone nano-capsules loaded with resveratrol (mean particle size 150 nm, PDI < 0.2, and encapsulation efficiency >80%) which are efficient in amorphization of resveratrol and thus improved solubility. With improved solubility, nano-resveratrol formulation significantly increased cytotoxicity in B16F10 melanoma cells compared to the resveratrol solution in in vitro study. In a mouse model bearing B16F10 melanoma tumors following 10 days intraperitoneal treatment (dose 5 mg/kg), the formulation showed decreased tumor volume (2807 mm^3^ in nanocapsule treated, 9656 mm^3^ in control and 7940 mm^3^ in resveratrol solution treated), increased necrotic area and inflammatory infiltrate of melanoma and thus prevented metastasis and pulmonary hemorrhage compared to the free resveratrol [107]. Palliyage et.al. [108] used the combination of curcumin with resveratrol and formulated solid lipid nanoparticles (diameter 180.2 ± 7.7 nm). The combination approach showed in vitro potential to stop metastasis in metastatic B16F10 cell lines based on electrical cell-substrate impedance sensing assay. Further the combination showed synergistic effect in inhibiting the growth of SK-MEL-28 cells [108]. Overall, formulations such as nano-emulsion, liposomes, dendrimers, nano-capsules, etc., have been proven to enhance the solubility and thus permeability of resveratrol across the skin.

## 5. Transdermal-Vaccine-Based Cancer Management

About 90% of cervical cancer is associated with the human papillomavirus (HPV) strains 16 and 18 [109]. Specifically, the E6 and E7 HPV proteins are involved in the malignant transformation of cervical cells via the suppression of the p53 and retinoblastoma protein (pRb), respectively [109]. Currently, the prophylactic HPV vaccines, Gardasil^®^ and Cevarix^®^, are given via the intramuscular route, target the E6 and E7 viral proteins [110,111]. Compared to the intramuscular route, the skin has a higher density of antigen-presenting cells (APC), such as Langerhans cells and dendritic cells [112]. The activation and maturation of APC induce T-cell-mediated interferon-λ secretion and activates CD8+ killer lymphocytes to decrease tumor infiltration [113]. Therefore, there is an emerging interest to deliver a vaccine via the transdermal route, using microneedles (MN). The viral proteins must retain their functionality when coupled into the MN array to elicit immunogenicity following transdermal administration. Kines et al. [114] showed, unlike regular vaccines, the production and distribution chain for MN-based vaccines are cheaper as they are stable at room temperature. They used the MN (stainless steel coated with 1% carboxymethylcellulose, 15% trehalose, and 0.5% Lutrol F-68 NF) containing lyophilized HPV-like particles (HPV–VLP) and HPV pseudovirions (PsV) composed of L1 and L2 capsid proteins of HPV16 and plasmids expressing the respiratory syncytial virus (RSV) antigens. In an in vivo study, the mice immunized with HPV PsV produced a neutralizing antibody response against HPV, resulting in a dose-dependent B- and T-cell response to the RSV antigen coded by the encapsulated DNA. Overall, the results suggested that HPV vaccines could be administered by using an MN approach [114]. Ali et al. [115] evaluated the utility of polymeric polyvinylpyrrolidone (PVP) based MN to deliver the RALA-E6/E7 NPs formulation in mice (50 μg via either intramuscular route or MN in-ear pinna). The antitumor efficacy achieved via the transdermal route was significantly greater than that of the intramuscular route. This was due to a high number of epidermal APC. Furthermore, RALA (a peptide with arginine/alanine/leucine/alanine repeated sequence), a novel 30 amino acid cationic peptide delivery sequence that forms nanoscale cationic particles by electrostatic interactions with viral DNA, preserves DNA’s functionality in the MN array. ELISA was used to quantify the E6/E7 specific IgG antibodies in mice sera where the IgG levels were two-folds higher in groups injected with NPs (both intramuscular and MN route) compared to control (naked DNA immunized). For prophylactic assay, the mice were first immunized with the RALA-E6/E7 NPs either intramuscular route or using MN then injected with TC-1 cells intra dorsally. The tumor remained undetected until 16 days in mice vaccinated with NPs of RALA-E6/E7 however in control group the tumor was palpable within seven days post-TC-1 injection. To check the anticancer activity mice with 50 mm^3^ of tumor were immunized with 100 μg of naked DNA or RALA-E6/E7 NPs via intramuscular or MN route. Complete regression was observed in 2/9 mice treated by using MNs, whereas it was observed in 1/9 mice treated via intramuscular route [115]. From the formulation aspect, in this same study, lyophilization in conjunction with MN fabrication technique enhanced the loading efficacy of RALA/pDNA NPs within the dissolvable PVA MNs. This lyophilized cake can be reconstituted into a small volume of aqueous MN matrix to increase the amount of drug loading in the MN [115]. By using lyophilization to increase the payload, Cole et al. [116] reported a significant increase in the in vivo delivery in the range of 50 μg of pDNA per MN array. Moreover, their results indicated that MN-based DNA vaccine delivery had greater efficacy than an intramuscular injection in a murine cervical cancer model [116]. Different materials are used in the synthesis of MNs, such as metal, solid silicon, and polymers [117]. The metal and silicon used in MNs need to be coated with the immunogenic antigen and this limits the drug loading capacity [118]. The polymers that dissolve in the interstitial fluid after insertion into the skin layers can overcome this limitation [118]. Cole et al. [119] tested the suitability of four different FDA-approved polymers based on PVP and PVA, such as PVP360, PVP-58, PVA-13-23, and PVA-9-10, for the transdermal delivery of nucleic acids. The recovery of the pDNA from the PVP polymer was poor, and this was due to the high concentration of the polymer used in the synthesis of the MNs. Although the supercoiled conformation of pDNA was changed in both PVA and PVP polymers, the transfection efficacy was maintained in the PVA matrices. The use of RALA in complexation with DNA prior to incorporation in the MNs was shown to be advantageous in PVP as this decreased biodegradation of pDNA and increased transfection efficacy. Overall, the PVA MNs were 10-fold more efficacious than PVP MNs [119]. Microneedles can be alternative route for vaccine delivery via transdermal route with better patient compliance and comparable efficacy.

## 6. Transdermal Permeation Study Using Selected Chemotherapeutic Agents

### 6.1. Five-Fluorouracil (5-FU)

5-fluorouracil (5-FU) is available in the form of a topical cream (0.5%, 1%, 4%, and 5%) and injection (50 mg/mL) for treatment of various cancers (i.e., colorectal, breast, cervical, melanoma, esophageal, etc.). It is bio-transformed to (1) fluorouridine triphosphate (FUTP), which competes with UTP for incorporation into RNA and affects its processing and function; (2) fluorodeoxyuridine triphosphate (FdUTP), which competes with dTTP for incorporation into DNA, causing DNA damage and (3) fluorodeoxyuridine monophosphate (FdUMP), which inhibits the conversion of deoxyuridine monophosphate to deoxythymidine monophosphate by inhibiting the enzyme, thymidylate synthase, thus decreasing the synthesis of DNA [120,121]. Overall, the above metabolites ultimately increase cancer cell death. However, it is important to note that only 1–3% of 5-FU dose is bio-transformed to the above-listed metabolites, which mediate its anticancer efficacy and produce the adverse effects associated with its use [122].

Pharmacokinetic studies in humans indicate that the oral absorption of 5-FU is poor [122]. In addition, due to hepatic metabolism, 5-FU has a terminal half-life of only 8–20 min. Thus, the transdermal route might achieve the delivery of 5-FU to the target site of action. Currently available topical creams are designed for local delivery within the skin layers for the treatment of basal cell carcinoma [123]. Thus, it is desirable to develop a transdermal formulation that can permeate across the skin in adequate quantities for the treatment of breast and other cancers. Lee et al. [124] assessed the efficacy of three different lasers to enhance the permeation of 5-FU across the skin. Laser ablation, a physical permeation enhancing technique, utilizes a laser to create channels on the SC layer of the skin [124]. The channels on the SC can be transient or persistent, depending on the laser pulse and time of exposure. A ruby, erbium:yttrium-aluminum-garnet (YAG) laser, and CO_2_ laser were used to enhance the permeation of 5-FU across the dorsal skin of the nude mice. The skin permeation was 5–10-fold greater with the laser than skin not exposed to lasers (control). Although the increase in flux (22-fold greater than control) across the skin was observed when the CO_2_ lasers were used, it produced thermal injuries. The results also indicated that when using the laser to ablate the SC, it was advantageous to use two lower pulses (0.8 J/cm^2^) instead of a single higher (1.4 J/cm^2^) pulse because the healing rate of skin after SC disruption is faster with lower pulses [124]. Huang et al. [125] formulated a microemulsion of 5-FU and evaluated the influence of the oil-phase, hydrophilic–lipophilic balance (HLB) value of surfactants, co-surfactants, and drug loading on the rat abdominal skin deposition. Of the three oils tested (i.e., sesame, canola, and ethyl oleate), canola oil produced the highest 5-FU permeation across the skin, whereas ethyl oleate produced a higher deposition in the skin. Importantly, canola oil has a higher amount of unsaturated fatty acids, which could facilitate skin permeation. In contrast, ethyl oleate has a long carbon chain, thereby explaining the higher entrapment of 5-FU in the skin instead of 5-FU permeating into the receptor fluid. The HLB of the emulsifier influences the globule sizes of the emulsion, a key factor in drug delivery and stability of the formulation [125]. For the microemulsion of 5-FU, the emulsion with HLB 6 produced the most efficient local deposition of drug in the skin layer. Ethanol and isopropyl alcohol were used as co-surfactants for the microemulsion of 5-FU, and isopropyl alcohol produced the highest drug localization in the epidermis compared to ethanol. Overall, the microemulsion consisting of ethyl oleate, isopropyl alcohol, and a mixture of surfactants with HLB 6.0, was shown to produce deposition of 5-FU in the skin similar to that of the commercial product, Efudix^®^ [125]. Raviraj et al. [126] demonstrated the use of superparamagnetic iron oxide nanoparticles (SPIONs) as chemotherapy adjuvants in transdermal drug delivery where SPIONs improved the transdermal delivery and antitumor activity of chemotherapeutic drugs in mouse melanoma. C57BL/6 mice subcutaneously injected with B16F10-tdTomato amelanotic melanoma cells were topically treated (3 times/week) with 1mM equivalent of 5-FU with or without SPIONs. Tumor volume monitored over 17 days showed significant reduction of tumor growth in group with SPIONs + 5-FU compared to 5-FU alone and vehicle treated. Further they also demonstrated enhanced permeation of DOX through the dorsal mouse skin with SPIONs compared to DOX alone [126].

The combination of certain chemotherapeutics, can treat cancers that have become resistant to monotherapy. For example, the use of curcumin and 5-FU formulation is shown to be efficacious in the treatment of cancer [127]. The oral route, however, has bioavailability limitations due to physiological barriers of absorption of the drug and hepatic metabolism. The use of a transdermal formulation could help overcome these limitations [128]. Although the transdermal drug delivery systems (TDDS) proved to be more effective compared to oral delivery, the advantages of combinatorial chemotherapy have rarely been extended to TDDS. Anirudhan et al. [129] fabricated nanoparticles containing 5-FU and curcumin entrapped in the hydrophilic and hydrophilic cores, respectively, these were composed of aminated nano-dextran (AND) and aminated β-cyclodextrin (AβCD). These active components were designed to leach out the drugs with varying kinetics as a function of the leaching solvent, which was selected based on comparable log-P values for curcumin and 5-FU. The in vitro permeation study showed an initial burst release for 24 h followed by a sustained release for 100 h. MTT assay was carried out in HCT116 cell lines (concentration range tested:1.5–50 ug/mL), where viability of 48.7% was reported [129]. In general, the transdermal route can be an alternative to circumvent the poor oral bioavailability issues of the 5-FU.

### 6.2. Doxorubicin Hydrochloride (DOX)

**Figure 5:**
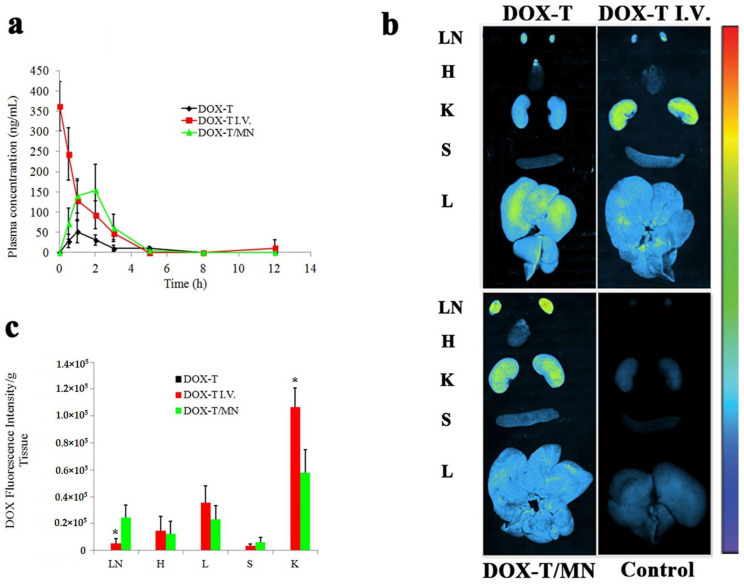
(**a**) DOX plasma concentration versus time profiles of intravenous injection (DOX-T I.V.) or transdermal administration with or without microneedles, labeled as DOX-T/MN or DOX-T, respectively (*n* = 5). (**b**) In vivo distribution of DOX-T, DOX I.V. and DOX-T/MN lymph nodes (LN), heart (H), kidney (K), spleen (S), and liver (L) of rats, respectively. (**c**) Quantitative distribution of DOX fluorescence intensity in rat organs (*n* = 5). * *p* < 0.05. Reproduced with permission from Yang et al. [134].

Doxorubicin hydrochloride (DOX) is an anthracycline ring-based anticancer drug, which is available in the form of injection (2 mg/mL) for treating lymphoblastic leukemia, myeloblastic leukemia, Hodgkin/non-Hodgkin lymphoma, and various metastatic types of breast cancer, lung cancer, neuroblastoma, soft tissue sarcoma, bone sarcoma, ovarian carcinoma, thyroid carcinoma and gastric carcinoma [130]. The DOX liposomal injection (2 mg/mL) is used for treating ovarian cancer, Kaposi’s Sarcoma, and multiple myeloma. DOX can produce significant cardiac toxic effects, which limits its therapeutic use [131,132]. Transdermal DOX delivery could decrease the incidence of cardiotoxicity and other adverse effects. Due to the high hydrophilicity of DOX, skin permeation is negligible. Therefore, MN-based transdermal systems of doxorubicin are gaining interest [133,134,135]. Unlike other physical techniques, such as ultrasound and thermal ablation, which are used to enhance permeation, MN does not require sophisticated instruments [32,136]. It is a painless physical permeation enhancement technique that disrupts the SC by creating an array of microchannels for the delivery of water-soluble compounds such as DOX [33]. Nguyen et al. [135] prepared polyvinylalcohol (PVA)-based MNs for DOX delivery, using a poly(dimethylsiloxane) (PDMS) mold. In a permeation study conducted across human cadaver skin, the PVA MNs were pressed against the dermatome human cadaver skin and left for 24 h. For the control, blank PVA-MNs were pressed against the skin, removed after 2 min, and drug solution (200 μL of 4.0 mg/mL) was applied over the skin. The results (flux 15.29 ng/cm^2^/h for drug localized in the tip of MNs) indicated that the localization site of the drug within the MN influences the release rate of the drug [135]. It is likely that the use of MNs in conjunction with drug solution would help achieve a rapid onset and adequate drug release. Yang et al. [134] formulated hyaluronic acid-based MNs that were integrated with DOX-loaded transferosomes. The skin permeation study (dose 67.5 μg/kg) indicated a 3-fold-higher bioavailability of DOX in rats when the MNs were used in conjunction with transferosomes compared to transferosomes alone. Further, a higher in vivo fluorescence intensity was observed in the lungs, liver, kidney, spleen, and intestines of rats that were given DOX with MNs integrated with transferosomes than the transferosomes alone and control group (Figure 5). Based on these results, the authors state that the MNs facilitated the transdermal delivery of DOX to the lymphatic system. Therefore, a transdermal approach that uses MNs could target cancer metastasis in tumor-draining lymph nodes [134]. Bhatnagar et al. [133] formulated composite MNs composed of PVP and PVA with a combination of chemotherapeutic drugs, i.e., DOX (50 mg) and docetaxel (DTX) (30 mg). The in vitro permeation study was carried out by using murine skin where 73.1% of DOX (367.4 ± 36.1 μg/cm^2^) and 26.7% of DTX (57.0 ± 5.6 μg/cm^2^) permeated through the skin over 48 h. Antitumor activity was compared between the control and group treated with a combination of DOX and DTX, using either intra-tumor injection or MN route (four doses each on day 1, 4, 7, and 10/equivalent dose via either route was given which was calculated based on the permeation profile of drug from MNs). The survival rates of athymic BALB/c mice with 4T1 breast cancer cell xenografts were significantly greater in mice treated with MNs compared to the mice that were given intra-tumoral injections of DOX and DTX. The intra-tumoral injection is labile to produce cardiac toxicity, myelosuppression, and mucositis associated with DOX and no animals survived following 9 days of treatment with two doses each of DOX and DTX. In contrast, when DOX and DTX were delivered by MNs, 100% of the animals survived following the administration of four doses until 16 days. The significantly lower magnitude of systemic toxicity after MNs-based transdermal delivery may be attributed to the slow release of DOX [133].

Carvalho et al. [137] evaluated the anticancer efficacy of colloidal-polysaccharide-drug nanocomplexes, made of carboxymethylcellulose (CMC)-DOX crosslinked with citric acid in a hydrogel-based formulation, using melanoma cell line (A375). The polymer-drug complex between DOX and CMC was achieved at pH 5.5, as the negatively charged carboxylate groups of CMC interacted with the positively charged amino group of DOX by strong electrostatic/ionic interactions. To facilitate the strong interaction required for complex formation, CMC was tailored with additional carboxylate groups on the cellulose backbone. The in vitro drug release from the hydrogel was pH–dependent, with a greater release of DOX at pH 6.2 compared to pH 7.4. This is important to target the acidic tumor microenvironment (extracellular pH in the malignant tumor is in the range of 6.5–6.9 [138,139]). In an MTT assay carried out by using HEK 293T (control) and A375 (melanoma cells), at early time points (6 and 24 h), the viability of A375 was lower compared to the HEK 293T cell line. It indicates the release of drug from the CMC–DOX complex specifically into the cancer-cell acidic microenvironment but not to the HEK 293T. In contrast, when free DOX was used instead of CMC–DOX, the viability of both control and melanoma cell line was reduced. Overall, the results of this study suggest that the electrostatic complexation strategy may be used to control the cytotoxicity of cancer drugs in transdermal formulations [137].

Overexpression of the ATP binding cassette (ABC) transporter, ABCB1 (also known as P-glycoprotein), which causes the efflux of DOX from cancer cells is one of the mechanisms by which melanoma cells become resistant to DOX [140,141,142]. The in vitro and in vivo resistance of certain types of cancer to DOX can be surmounted by drugs such as trametinib (Tra) that inhibit the binding of intracellular DOX to the ABCB1 transporter [143,144,145]. BRAF-mutant melanoma proliferates and survives primarily by activation of mitogen-activated protein kinase kinase (MEK) [146]. Trametinib (Tra) inhibits MEK1 and MEK2 kinase activity and had shown promising effects in the management of metastatic melanoma in clinical trials [147]. Huang et al. [148] formulated MNs by using the novel polymer, dextran methacrylate (DexMA), using a combination of drugs, i.e., DOX and trametinib. DOX is a water-soluble drug, whereas trametinib is a lipophilic drug. This study demonstrates the feasibility of using DexMA polymer for the formulation of molecules with varied physiochemical characteristics. In an in vivo study B16 xenograft, nude mice model was treated with MNs (DOX dose of 50 μg and Tra dose of 25 μg, the patch was placed over the tumor and replaced every day), control (without drugs), a group given tail vein injection of DOX (50 μg) and group given oral Tra (25 μg). The tumor suppression in a group treated with MNs of DOX-Tra was significant (*p* < 0.01) as indicated by smaller tumor size and lighter tumor weight. In addition to that, the weight of the DOX injected group falls from 14 to 11 g whereas weight was consistent in MNs treated mice. Loss in weight demonstrates the possible toxic effect of DOX through IV route whereas MN based transdermal route seemed to be a safer option for DOX delivery [148]. Ahmed et al. [149] formulated liposomes possessing DOX in combination with celecoxib and used a Derma roller^®^ to enhance the transdermal permeation of the formulation. The in vivo study was carried out by using xenograft mice with B16 murine melanoma tumor, and the antitumor efficacy of DOX and Celecoxib liposome gel (200 mg gel with 2 mg/kg DOX and 10 mg/kg celecoxib applied for 8 consecutive days) with or without microneedles was compared. The tumor size was significantly smaller in the group where a Derma roller^®^ was used compared to the group without a Derma roller^®^. The reduction in tumor volume with DOX, DOX + MN, DOX + celecoxib, and DOX + celecoxib + MN was found to be 2280.473 ± 151.44 mm^3^, 1759.75 ± 310.23 mm^3^, 1194.53 ± 166.24 mm^3^, and 597.76 ± 194.06 mm^3^, respectively. The average tumor weight and the weight of tumors in the groups treated with derma roller prior to gel application were smaller than the untreated groups. Further, the weight of the animals (20 g) did not change significantly after the treatment period indicating a better toxicity profile of the transdermal delivery. Hematoxylin–eosin (H&E) staining showed that tumors that were pretreated with MNs resulted in smaller loosely distributed melanoma cells with wide intercellular spaces when compared with MNs untreated groups (control and DOX groups) [149]. Nguyen et al. [150] studied maltose MN arrays for transdermal delivery of DOX solution across human cadaver skin. The skin was pretreated with MNs, using a spring applicator, and two doses of DOX (0.2 and 0.4 mg) were applied. The permeation of DOX across the skin was significantly higher in the MN pretreated skin compared to untreated skin [150].

A study reported the use of carbon nanotubes (CNT) for transdermal delivery of DOX (hydrophilic model molecule) and indomethacin (hydrophobic model molecule) [151]. The CNTs provide surface where the drug can be loaded or adsorbed for delivery [152]. The specific permeation route of CNTs is yet to be elucidated, but it has been hypothesized that CNTs permeate by passive diffusion across the lipid bilayer [151]. CNTs can produce dermatitis and hyperkeratosis due to their entry and entrapment in the skin layers [153]. The double-walled carbon nanotubes (DWCNTs) (Nanocyl 2151) and multi-walled carbon nanotubes (MWCNTs) (Nanocyl 3101) were evaluated for their drug adsorption capacity in which MWCNTs were found to be more efficient in adsorbing the drug compared to DWCNTs. MWCNTs were further functionalized with PEG 3350 to evaluate the drug dispersion ability where the PEGylated MWCNTs demonstrated low drug adsorption compared to non-PEGylated form. Further, the permeation of adsorbed drug across the pig-ear skin from nanotubes was investigated. The passive permeation of DOX from MWCNTs was low because of their strong adsorption limiting the amount of free drug that could permeate through the skin. However, the MWCNTs can be used as an adsorptive electrode, where the permeation of DOX through the application of iontophoresis is markedly higher [151]. The controlled permeation of DOX across the skin can be achieved by using another physical permeation technique, electroporation, with non-invasive electrodes (multi-electrode array). Blagus et al. [154] demonstrated controlled in vivo permeation of DOX (100 μL in a dose of 10 mg/kg) across the mice skin by using electroporation at 360 and 570 V pulse amplitudes. The non-invasive electrode facilitated topical or transdermal delivery by varying the amplitude of the electric pulse without damaging the skin. The vascular effects (increased permeability of the endothelial lining and extravasation of molecules in microvessels) in mice were dependent on the threshold and the amplitude of electric pulses. For example, after the pulse exceeded 360 V, vasoconstriction occurred in the tumors, causing a delay in perfusion, whereas an increase in pulse amplitude increased the permeability and cellular drug uptake [154]. Zhang et al. [155] designed a transdermal nanoplatform (+)T-SiDs of DOX, consisting of super-paramagnetic iron oxide (SPIO) with cationic phospholipids, transdermal enhanced peptide (TD), 1,1′-dioctadecyl-3,3,3′,3′-tetramethylindotricarbocyanine iodide (DiR). This system was used for targeting superficial tumors via the transdermal route. Using 4T1 breast cancer cells, they demonstrated high photo thermal conversion efficiency and stability, efficient cellular uptake, synergistic cancer-cell-killing effect, and enhanced percutaneous permeability in vitro. Further (+) T-SiDs administered via transdermal route displayed superior tumor inhibition and higher biocompatibility compared to the intravenous route in 4T1-tumor-bearing mice [155].

To summarize, the transdermal route subdues the cardiac toxicity associated with DOX. Although DOX is highly hydrophilic, the use of appropriate formulation with permeation strategies, such as MNs, electroporation, iontophoresis, etc., can make it a potential candidate for transdermal delivery.

### 6.3. Methotrexate (MTX)

Methotrexate (MTX) is used to treat acute lymphocytic leukemia, breast cancer, lung cancer, non-Hodgkin lymphoma, osteosarcoma, and CNS embryonal tumors [156]. It is a folic acid analog that inhibits the enzyme dihydrofolate reductase, which ultimately inhibits the synthesis of tetrahydrofolate, required for the synthesis of purines, pyrimidines, serine, and methionine, that are critical for the Novo synthesis of DNA [157]. MTX is also used in the treatment of psoriasis and rheumatoid arthritis [158]. MTX is available as a subcutaneous injection (25 mg to 62.5 mg/mL), MTX sodium is available as an injection (25 mg/mL as a base) and as an oral solution (25 mg/mL as a base). The oral, subcutaneous, and intramuscular administration of MTX, depending on the dose and length of treatment, can produce severe toxic effects, such as hepatotoxicity, leukopenia, anemia, thrombocytopenia, and GI bleeding [156]. Therefore, the transdermal route can be a viable approach to circumvent the toxic effects produced by the systemic administration of MTX. MTX is a hydrophilic drug and hence has low skin permeability. This has led to the development of various novel transdermal formulations containing MTX. Zeb et al. [159] prepared ultra-deformable liposomes (UDL) with MTX, containing phosphatidylcholine in the bilayer matrix and sodium cholate or Tween 80 as the edge activator. UDL can transform their shape and volume at a lower energy due to their high curvature radius and mobile edge activators. Upon exposure to stress, the edge activator can relocate to the zone of high curvature, whereas the phospholipids are positioned in the bilayer regions, due to their smaller curvature [159]. The transformation of the shape and volume of the UDL allows them to pass through the pores smaller than their size without losing their vesicular structure. The UDL (with a 7:3 ratio of phosphatidylcholine: Tween 80 *w/w*) dispersed in Carbopol gel had shown 1.5- and 2.15-fold higher permeation of MTX across the abdominal skin of rat compared to liposomes with cholesterol and plain Carbopol gel [160]. The combinations of more than one physical permeation technique, such as iontophoresis + sonophoresis, iontophoresis + MNs, and MNs + fractional ablative laser, have shown improved transdermal delivery of MTX [161,162,163]. The micro-channels formed across the skin by lasers are significantly larger than those formed by microneedles [163]. One study has shown significantly higher permeation of MTX across the skin following sonophoresis compared to iontophoresis [162]. Physical permeation-enhancing techniques can compromise skin integrity, and this can be monitored by measuring the electrical resistance across the skin layer [164]. The altered skin integrity due to iontophoretic transdermal delivery of MTX at 0.2 mA/cm^2^ was reversed within 48 h [165]. However, when the current was increased to 0.5 mA/cm^2^, irreversible histological changes occurred in the skin, such as appendageal dilations and focal disruption of the epidermis [165]. Yang et al. [158] prepared a magnesium oil (MO)-enriched MTX nano-emulsion (175 ± 35.4 nm with EE 65 ± 8.6%) which was gelled by using Carbopol 940 for the transdermal delivery of MTX. In an in vivo study involving rats with arthritis, the arthritis score and paw volume in the group treated with MTX-MO nano-emulsion gel was significantly lower than the control [158]. The use of surfactants to enhance transdermal permeation has been reported for various formulations of MTX. For example, Javadzadeh et al. [166] determined the effect of three different surfactants, cationic, anionic, and non-ionic, at varying concentrations, on the permeation of MTX across the rat skin. Sodium lauryl sulfate (SLS) and benzyl dimethyl chloride did not significantly enhance the percutaneous permeation of MTX. In contrast, the non-ionic surfactant, Transcutol^®^ (2% *w/w*), along with salicylic acid (6% *w/w*) enhanced the transdermal permeation of MTX.

### 6.4. Paclitaxel (PTX)

Paclitaxel (PTX) is a natural product with antitumor activity. It is obtained via a semi-synthetic process from *Taxus baccata*. It has a molecular weight of 853.9 Da. This drug is used to treat ovarian cancer, non-small-cell lung cancer, and breast cancer. It is also used to treat Kaposi’s sarcoma. PTX is available as injectable suspension (albumin-based nanoparticles containing 5 mg/mL of PTX after reconstitution), and as an injection (6 mg/mL). Transdermal delivery of this drug will be beneficial in terms of avoiding hospital visits for injections and achieving local delivery in breast cancer avoiding dose-related side effects. Panchagnula et al. [167] investigated the possibility of enhancing the transdermal delivery of PTX with the aid of permeation enhancers, fatty acids, and terpenes. The SC, which contains a rigid lamellar lipid layer, forms a barrier that limits the transdermal delivery of drugs [168]. Fatty acids are used as permeation enhancers, which increase the fluidization of the SC lipid bilayer and the formation of the fatty acid pool. Terpenes with hydrogen bond donors or acceptors, such as menthol and menthone, can decrease the integrity of the SC bilayer by disrupting the formation of hydrogen bonds between ceramides, thereby increasing PTX permeation [169].

The systemic administration of PTX can be used to treat HIV-associated forms of Kaposi’s sarcoma and basal cell carcinoma [170,171]. However, the systemic use of PTX for the treatment of skin cancer is limited by neutropenia, hypersensitivity reactions, and thrombocytopenia [172,173]. To circumvent the systemic toxic effects of PTX, researchers have prepared topical formulations of PTX for potential use in treating basal cell carcinoma. Hosmer et al. [174] utilized liquid crystalline phases (LP) based on polyoxyethylene-10-oleoyl ether (BRIJ 97) containing medium-chain mono/diglycerides (MCG) as a topical delivery system for PTX. BRIJ is a hydrophilic surfactant that forms a variety of structures upon self-assembly, such as isotropic micro-emulsions and LP and hexagonal phases (HP), depending on the temperature and water content [174]. The MCGs were incorporated at different concentrations in BRIJ to enhance the permeation of PTX through the porcine ear skin without interfering with the LP and HP phases of BRIJ. The LP produced a greater topical delivery of PTX compared to the HP, which might be attributed to the higher water content and higher viscosity of HP, which limits the diffusivity of PTX, a lipophilic drug. Furthermore, within the LP, MCG concentration-dependent retention of the PTX in the skin occurred at a higher concentration of MCG (20% or higher). In contrast, low concentrations (10% or lower) of MCG in the LP favored transdermal permeation [175]. The influence of the length of the glyceride acyl chain added to LP to optimize the balance between the cutaneous and transdermal delivery of PTX was also investigated by Hosmer et al. [174] Monoglycerides, with acyl chain lengths ranging from 8 to 18 carbons (monocarprylin, monomyristolein, and monoolein) at a concentration of 20% were used in the study. There was an inverse relationship between the length of the monoglyceride acyl chain and topical delivery of PTX. The increased skin accumulation of PTX after the incorporation of monoglycerides in the formulation could result from the affinity of PTX to the monoglycerides that were partitioned in the skin layer [174]. Utrejal et al. [176] formulated PTX as elastic liposomes, consisting of phospholipids and surfactant (span 80). The drug loading in this formulation was 6.0 mg/mL. The elastic liposomes produced significant steady-state transdermal flux and accumulation in the skin based on in vitro permeation studies, using rat abdominal skin [176]. Furthermore, the local application of PTX for breast cancer chemotherapy in the absence of surfactants such as Cremophor EL can be a promising approach to reduce the incidence of adverse effects such as hypersensitivity [177].

## 7. Formulating for Efficacy™ (FFE)

There are only a few software programs available for the in silico optimization of transdermal formulations. Software’s such as Creativity Formulation Software™, Smart Formulator™, Formpak™, etc., are upgraded formulation spreadsheets that allow researchers to maintain raw material database s, prices as well as help to create and edit formula sheets [178]. There are other alternative software programs that take into account of active pharmaceuticals. However, these in silico options, such as BIOiSIM™, focus more on pharmacokinetic modeling of drugs rather than excipients or excipient optimization [179]. So far, from our knowledge, the only currently available software that aims to optimize both the drug and excipient in silico is Formulating for Efficiency™.

Formulating for Efficacy™ is a software initially created to address active delivery concerns in cosmetics, using a theoretical approach [180]. Formulating a product that achieves the intended, active delivery result can be expensive and time-consuming. When formulating a product, the prototype is typically not successful. Therefore, formulators troubleshoot their formulas until they obtain desired results. This process can take multiple trials that ultimately translates to a loss of money and time. Having software such as FFE that can predict optimized formulas for an active of interest can decrease significant amount of work in the formulating process and increase productivity as well as laboratory efficiency.

To use FFE, active ingredients and excipients are entered by using their Simplified Molecular Input Line Entry Specification (SMILES) code, and the software then generates their solubility and physicochemical properties. These properties include but are not limited to the ingredient active gap (IAG), active formulation gap (AFG), ingredient skin gap (ISG), molar volume (MVol), and Hansen solubility parameters (HSP; δD, δP, and δH values) [181]. FFE is only set up for optimizing lipophilic actives, yet it is still possible to run a simulation with a hydrophilic active. Different molecules can be added to the software but currently, there are limitations for the addition of salts (those containing two ionic species are not recognized) and polymers. Creating a mixture of two ingredients is possible by using the “Mixed Ingredients Calculator”. Any ingredient with a high molecular weight, such as emulsifiers and polymers, should not be included in the software as these high molecular weight compounds are argued not to contribute to permeation. There is a three-dimensional option that results in a pop-up screen depicting Hansen Space (δD, δP, and δH), which shows up as a green sphere for the skin, a blue sphere for ingredients, a yellow sphere for actives, and a pink sphere for the formulation. This allows for a visual comparison of HSPs [182].

The software user can choose to model the excipients of their choice in the required percentages. After selecting the ingredients in the required percentages one can calculate the HSPs, SFG (Skin Formulation Gap), and AFG of the formulation [182]. Formulators also have the option to allow FFE to choose the best two or three excipients optimized to the active, skin, or target concentration. If a formulator chooses to have more than three excipients in the oil phase, there is also an option to find an additional ingredient by selecting “find best extra ingredient for current formulation”. Optimizing to the active will match the active with the excipient HSPs, thus allowing a low-solubility drug to dissolve to the highest extent possible [182]. Optimizing to the skin will match the HSPs with the SC and ensures that the active is capable of penetrating the skin layers. Finally, optimizing to the target concentration ensures that the active concentration is near its solubility limit [182]. With these options, the software also provides the percentages of the excipients at which the formulation is optimized, given the total oil phase is entered. The optimal selection of the excipients in the formulation is based on the Relative Polarity Index which accounts for the polarity of the active, oil phase, and SC [183]. However, FFE does not take into account the maximum allowable use level for each excipient [182]. Therefore, it is important to account for these possibilities for FFE to select the best excipients. Formulators can then calculate the solubility of the active in the formulation (SolV) and the solubility of the formulation in the skin (SolS).

There is also a feature that can determine the ratio between the Minimum Effective Concentration (MEC) based on cell culture studies performed and the Local Tissue Concentration (LTC). The MEC should be manually entered, and the LTC can be calculated by using two approaches: the Classic Flux Method and the FFE Flux Method. The Classic Flux Method utilizes Fick’s law of diffusion and assumes that the flux going into and out of the skin is the same. The equation used to calculate this is as follows: LTC = (kp · ΔC)/Cl, where k_p_ is the permeability coefficient, Cl is clearance, and ΔC is the concentration gradient of the drug. The software lists both the molecular weight (MW) and Log K values so that k_p_ can be calculated by using the Potts–Guy equation. In addition, users should measure the saturated aqueous concentration of the active and enter it in the appropriate box. The limitations with such a theoretical approach are that it assumes the clearance of actives is equal to that of the blood flow and that the actives are equally distributed in all the layers of the skin. However, the FFE Flux Method has been proposed to be a more targeted approach since it utilizes the parameters calculated from the actual formulation. It uses Fick’s law of diffusion and bases the length of the pathway on the thickness of the SC. It also incorporates the thickness of the dermis. The flux value given is that of the flux into the dermis [182]. This formula can also be entered in the Diffusion Modeler, which gives hypothetical Franz diffusion cell results (for short or sustained-release studies). The user can set a simulation time for their formula through the modeler screen. Parameters such as surface concentration, permeation rate, and total active in, on, and out the skin can be obtained for the set simulation conditions.

Despite being created to address cosmetic formulations, FFE has recently been used to address drug formulations [183,184]. In a study titled “Optimized transdermal delivery of pravastatin,” researchers formulated three creams and three emulgels with 2% pravastatin. Two of these formulations (a cream and emulgel) were optimized by using FFE, while four other formulations were created to be polar or non-polar. The results indicated that the optimized formulations were more widely diffused through the SC and into the target site of Caucasian full-thickness abdominal skin in a Franz cell assay [184]. Another study was done comparing a marketed ibuprofen emulgel to formulations optimized by FFE. A Franz cell study using both porcine ear skin and a Strat-M membrane indicated that the 24-h permeation of the ibuprofen formulations optimized by FFE were significantly greater than the marketed product [183].

## 8. Conclusions

The transdermal approach of chemotherapeutic drug delivery has been recognized for its advantage over the oral and parenteral routes in different cancer types. A major limitation of the transdermal approach is related to the low drug permeability across the SC to achieve the plasma concentration required for therapeutic efficacy. However, a majority of chemotherapeutic drugs are very potent and are effective at low doses. Therefore, the design of chemotherapeutics agents as transdermal dosage forms can be a promising approach. In addition to that, for few cancer conditions, such as breast and skin cancers, the tumors can be intervened by the local application of drugs to the skin over the hot spots, which will not only deliver the drug to the target site but also substantially reduce the systemic toxicities of the cytotoxic drugs.

Here we summarized the popular chemotherapeutics formulation strategies used for targeting the local tumors primarily focusing on the skin and breast cancer. Topical emulsions could be used to incorporate both hydrophilic and hydrophobic drugs, in the water and oil phase, respectively. Nano-emulsions can be useful to overcome the stability issues of regular emulsions. The drugs that are intended for sustained release, such as letrozole, can be formulated in the form of transdermal patches. The transdermal patches in conjunction with microneedles can enhance the drug permeation across the skin to achieve the target therapeutic dose. For the drugs that are highly hydrophobic in nature, such as paclitaxel, drug carriers, such as liposomes, micelles, and nanoparticles, are well suited for transdermal delivery. Carrier systems such as ethosomes and niosomes are suitable for hydrophilic drugs. In addition to the physiochemical characteristics of the drug, the excipients of the formulation influence the drug loading. For instance, in PLGA nanoparticles preparation, the higher percentage of lactic acid would favor higher entrapment of relatively hydrophobic molecules such as paclitaxel, whereas a higher percentage of glycolic acid favors the entrapment of hydrophilic molecules such as doxorubicin. Similarly, PLGA end-capped carboxylic group favors higher entrapment of polar drugs compared to the one without carboxylic group. The appropriate choice of excipients in the formulation design is based on the physiochemical nature of the drug, and this is pivotal for the successful design of a formulation.

The advancement in nano-carrier systems has revolutionized the field of drug delivery, which has positive implications in transdermal delivery as well. The carrier systems used in transdermal delivery, such as liposomes, nanoparticles, micelles, dendrimers, etc., facilitate the delivery of drugs with diverse physiochemical properties. These carrier systems support the combination approach, allowing the simultaneous delivery of two or more drugs in a sustained release pattern. Further, the physical permeation-enhancement techniques, such as microneedles, sonophoresis, iontophoresis, etc., have expanded the delivery of diverse drug classes (both small molecules and large molecules) via transdermal route. The transdermal formulation for the management of cancer is yet to be optimized, and safety concerns are yet to be addressed from the regulatory standpoint. Some limitations of transdermal chemotherapeutic delivery include poor drug loading, stability of the formulation, inadequate permeability of hydrophilic compounds, and high dose requirements to exert therapeutic effects. Although the transdermal route can target superficial tumors, it might not be an ideal approach when the tumors have grown and metastasized and in the management of internal organ cancers. The optimization of the formulations through the design and the establishment of a safety profile are prior requirements for clinical translation of the transdermal chemotherapeutics. The advent of transdermal chemotherapeutics in clinics would improve patient compliance compared to parenteral administration.

The design of the transdermal dosage form can be challenging depending on the chemistry of the molecules. The selection of appropriate excipients via the hit and trial method is rigorous and filled with uncertainty. Therefore, using in silico models that could predict the result of the transdermal formula would be an effective way to save time and resources. Here we introduced the use of Formulating for Efficacy™ for the design of chemotherapeutic dosage forms. Although the use of this software is only recently being explored, the development of such in silico software would aid a lot in dosage form design in the near future.

## Figures and Tables

**Table 1:** Ideal physicochemical and pharmaceutical properties for passive transdermal drug delivery [2,20].

Critical Properties	Ideal Limits
Aqueous solubility	>1 mg/mL
Lipophilicity (log octanol/water P)	>1 and <4
Molecular weight	<500 Da
Melting point	<200 °C
pH of the saturated aqueous solution	5–9
Daily dose	<20 mg
Skin irritation or sensitization	None

## Data Availability

Not applicable.
